# Sexual risk related behaviour among youth living with HIV in central Uganda: implications for HIV prevention

**DOI:** 10.11604/pamj.2016.24.49.6633

**Published:** 2016-05-11

**Authors:** Racheal Ankunda, Lynn Muhimbuura Atuyambe, Noah Kiwanuka

**Affiliations:** 1 Research Department, Ernest Cook Ultrasound Research and Education Institute, Mengo Hospital, Kampala, Uganda; 2 Makerere University School of Public Health, MPH Program Alumni, Makerere, Uganda; 3 Makerere University School of Public Health, Makerere, Uganda

**Keywords:** Sexual risk related behavior, abstinence, youth living with HIV/AIDS, HIV prevention

## Abstract

**Introduction:**

As young people living with HIV grow their sexual behaviour and it's implication on HIV prevention is of concern. This study describes the sexual risk related-behaviours and factors associated with abstinence among Youth Living with HIV in central Uganda.

**Methods:**

We conducted a cross-sectional study among 338 unmarried youth between 15 and 24 years accessing HIV care in central Uganda. Data was collected using interviewer administered structured questionnaires. Adjusted prevalence proportion ratios (adj. PPRs) of factors associated with sexual abstinence for at least six months were determined by multivariable log-binomial regression.

**Results:**

Overall, 79% (269/338) of respondents were abstaining from sexual intercourse for atleast six months, although, 45% (150/338) had ever been sexually active. Of the 283 respondents who desired to get married in future, 40% preferred negative marriage partners. Only 31% (39/126) of respondents in boy/girl relationships had disclosed their HIV status to their partners. Among those currently sexually active (n = 69), 57% did not consistently use condoms and 30% had more than one sexual partner in the past six months. The adj.PRR of abstinence was higher among youth between 15 and 19 years compared to those between 20 and 24 years (adj. PPR = 1.26, 95% CI; 1.08-1.46). The prevalence of abstinence was significantly lower among respondent who consumed alcohol (adj. PPR = 0.31, 95% CI 0.16-0.61).

**Conclusion:**

Tailored interventions promoting disclosure, consistent condoms use and discouraging alcohol consumption among sero-positive youth could reduce HIV transmission risk.

## Introduction

Globally, one third of new HIV infections among individuals above 15 years occur among youth between 15 and 24 years [1]. Majority of these new infections occur in sub-Saharan Africa and female youth carry a disproportionately greater burden of HIV infection [2]. The prevalence of HIV among youth is growing due to new infections among the youth and maturation of perinatally infected children due to increased access to antiretroviral therapy (ART) [3]. With increasing HIV prevalence among the youth, HIV prevention intervention tailored to Youth Living with HIV (YLWH) are critical in controlling the epidemic. In Uganda, the number of YLWH is rapidly increasing with an HIV prevalence of 3.7% among individuals between 15 and 24 years. HIV prevalence is distinctly higher among young women than men [4]. Similar to other youth, YLWH begin to explore their sexuality early in life [5]. According to the 2011 Uganda AIDS Indicator survey, 15% of youth between 20 and 24 years have had sex by 15 years [4]. HIV prevention interventions for YLWH emphasize delaying sexual intercourse, reducing the number of sexual partners and using condoms [6]. YLWH experience unique challenges because of the intricate relationship between sexuality and HIV transmission [7]. It is therefore recommended that prevention intervention addressing their specific sexual and reproductive health desires are identified and implemented within a positive prevention framework which aims to protect sexual health, avoid sexually transmitted infection, delay HIV/AIDS disease progression and avoid onward HIV transmission [8]. As YLWH get older, they desire to have sexual intercourse, get married and bear children [9], sexual initiation and its implication on HIV transmission is a concern. Despite preventive support and counselling, findings from central Uganda reveal that the sexual behaviour of some sero-positive youth may place others at risk of HIV acquisition [10]. Unsafe sexual practices predispose their uninfected partners to a serious risk of HIV acquisition. There is limited information on sexual practices and other HIV transmission related behaviours of YLWH, most studies conducted focus on either adolescents between 10 and 19 years [9, 10] or on adults above 18 years [11, 12]. These findings cannot be adequately generalized to youth between 15 and 24 years. Following a literature review, we conceptualized various socio-demographic, health related and behaviour factors that possible affect sexual behaviour of YLWH. Using the variables in this conceptual framework, we assessed the sexual risk related behaviour and factors associated with abstinence among YLWH. In this article we report on the sexual practices, partner preferences and factors associated with abstinence among YLWH.

## Methods

### Study site

Participants were recruited from Nsambya Home Care Department in Kampala district and Mildmay Center in Wakiso district. Mildmay and Nsambya Home Care Department are both independent, non-governmental, not-for-profit, Christian founded organizations offering HIV care to children and adults from mainly the urban and peri-urban areas of central Uganda. Services offered at the study sites include; HIV testing and counselling, out-patient medical care for sero-positive individuals, provision of antiretroviral therapy and psychosocial support.

### Sample size estimation

Using Leslie Kish's formula for descriptive cross-sectional studies [13] to determine sample size, we estimated the prevalence of abstinence among seropositive youth at 50%. Using a precision of 5% and adjusted for a 10% non-response rate a sample size of 427 approximately 430 participants was computed.

### Study design and sampling procedures

Between January and March 2010 we conducted a cross sectional study to assess the sexual behaviour and factors associated with abstinence among youth living with HIV. A three months time frame was deemed appropriate for data collection since the maximum duration between each clients appointment was three months depending on how stable the client was or if they were if they were attending boarding school. It was therefore anticipated that majority of youth between 15 and 24 years would have attended atleast one clinic appointment within this three months data collection period. Youth accessing HIV/AIDS care at Mildmay Centre and Nsambya Home care department, who at the time of data collection had known their HIV status for a period greater than nine months were included. Only clients attending a routine follow up appointments were included in this study, those presenting for treatment of acute illnesses or who were sick/too weak at the time of their scheduled appointment were not included. The total number of registered clients between 15 and 24 years at Mildmay and Nsambya Home Care was 1,415 (1,033-Mildmay and 382-Nsambya home care department). Using proportionate sampling 314 respondents were sampled from Mildmay and 116 respondents sampled from Nsambya Home Care Department. At each of these facilities youth between 10 and 18 years had special adolescent clinic days, Tuesday for Nsambya Home Care and Thursday for Mildmay. The older youth attended adult clinic days. For equal representation, half of respondent interviewed were 18 years and below and the other half were 19 years and above (Mildmay 314/2 = 157, Nsambya Home care 116/2 = 58). During the study period, Clients between 15 and 18 years were enrolled on the adolescent clinic days while those who were 19 and above were enrolled on the adult clinic days-two data collection day were designated each week at each site. There were 12 data collection days for either adolescents or adults over the three months period. In Mildmay, 157 adolescents below 19 years and 157 adults from 19 years were sampled. Similarly in Nsambya home care 58 adolescents and adult clients were sampled. Using systematic sampling, 13 youth (157/12) below 19 years and 13 youth from 19 years and above were sampled at Mildmay on each of the 12 data collection days. Five (58/12) youth were sampled on each of the adolescent and adult clinic day in Nsambya. The sampling frame varied because the number of clients expected varied on each adolescent clinic day. During the study period, eligible clients attending the adult clinic were screened at the registration table, given information about the study, consented and consecutively enrolled. Of the 430 youth sampled, 425 (98.8%) provided written consent and were enrolled into the study. It ought to be noted that of the 425 respondents enrolled, 87 were not included in this analysis because they were married, leaving a sample size of 338 respondents.

### Data collection

Data was collected using a structured questionnaire administered by same-sex interviewers who spoke both English and the local language (Luganda). The data collectors were not affiliated to the study sites and were trained for three days on the study objectives, sampling techniques and methods of data collection. Interviews were conducted in the health facility compound away from other services to ensure privacy. Only respondents were present for the interviews which lasted between 30 to 45 minutes.

### Data management and analysis

The completed questionnaires were checked for consistency and completeness on the day of data collection and errors were corrected in the field. Questionnaires were entered into EPIDATA^®^ software and data was exported to STATA^®^ version 12.0 for analysis. The general characteristics of the study population were summarized using means (SD) and medians (IQR) for continuous variables and proportions for categorical variables. Participants’ characteristics were compared using t-tests for continuous variables and chi-square tests or fisher exact tests for categorical variables. The outcome variable-sexual abstinence was defined as refraining from penetrative sexual intercourse for at least six month before the study. Owing to the high prevalence of abstinence (79%), we used log-binomial regression with log family and poisson link to estimate the unadjusted and adjusted prevalence proportion ratios (PPRs) and corresponding 95% confidence Interval (95% CI) of factors associated with abstinence among YLWH. Covariates that were significant at p < 0.05 at bivariate analysis and those with biological plausibility were included in the multivariable log binomial regression with log family and poisson link. The final adjusted model included abstinence as the outcome and the following predictor variables: HIV care site, sex, age, staying with parent/guardian, having biological children, route of HIV acquisition and alcohol consumption.

### Ethical considerations

Ethical approval to carry out the study was obtained from the Makerere University School of Public Health Higher Degrees, Research and Ethics Committee and University Hospital Case Medical Center-Institutional Review Board.

## Results

### Characteristics of study respondents

Table 1 describes the characteristics of the study population stratified by HIV care site. Of the 338 participants, 72% (242) were females, the mean (SD) age was 19 (3.3) years, 86% (291) were single. Forty five percent (45%) of respondents were out of school and 63% had attained secondary school education and above (tertiary school/institution education). Seventy three percent (247) did not have a biological child. Most respondents, 81% (274) were on antiretroviral therapy and 78% reported that they had not missed a clinic appointment in the past six months. Of the respondents interviewed, 247 (76%) were from Mildmay and 24% from Nsambya Home Care Department. Majority of youth in both sites were female. The two populations were similar in terms of marital status, school attendance status, residing with parents/guardians, child bearing history, adherence to clinic appointments and perceived route of HIV acquisition. There was no difference in the mean age of respondents from the two populations and mean age of first knowing their HIV status. Respondents’ level of education, enrolment on antiretroviral therapy and membership in a peer support group were significantly higher among youth from Mildmay centre. Alcohol consumption was significantly higher among youth from Nsambya home care (chi-square p value = 0.044).

**Table 1:** Characteristics of study population stratified by HIV care site

Variable	Total (n = 338)	Mildmay Centre (n = 257)	Nsambya home care department (n = 81)	p value
**Mean age in years (S.D) of;**				
Respondents	19.0 (±3.28)	19.6 (±3.22)	19.2 (±3.49)	0.561^a^
Knowing HIV status	15.1 (±4.86)	14.9 (±4.38)	15.9 (±4.86)	0.088^a^
**Age Groups**				
15-19	204 (60.4%)	157 (61.1%)	47 (58.0%)	0.623
20-24	134 (39.6%)	100 (38.9%)	34 (42.0%)	
**Sex**				
Female	242 (71.6%)	181 (70.4%)	61 (75.3%)	0.396
Male	96 (28.4%)	76 (29.6%)	20 (24.7%)	
**Current marital status**				
Single	291 (86.1%)	226 (87.9%)	65 (80.2%)	0.081
Separated/Widowed	47 (13.9%)	100 (12.1%)	16 (19.8%)	
**Religion**				
Catholic	126 (37.4%)	95 (37.1%)	31 (38.3%)	
Protestant	95 (28.2%)	70 (27.4%)	25 (30.8%)	
Moslem	53 (15.7%)	40 (15.6%)	13 (16.1%)	
Born Again	63 (18.7%)	51 (19.9%)	12 (14.8%)	
**Education level**				
Low (No education & Primary)	112 (33.1%)	76 (29.6%)	36 (44.4)	0.013^+^
Moderate (Secondary & Tertiary)	226 (66.9%)	181 (70.4%)	45 (55.6)	
**School Attending**				
Out of school	151 (44.7%)	111 (43.2)	40 (49.4%)	0.328
Student	187 (55.3%)	146 (56.8)	41 (50.6%)	
**Staying with Parent/guardian**				
Yes	283 (83.7%)	218 (84.8%)	65 (80.3%)	0.33
No	55 (16.3%)	39 (15.2%)	16 (19.7%)	
**Child bearing history**				
Has biological child	91 (26.9%)	65 (25.3%)	26 (32.1%)	0.228
Has never had a child	247 (73.1%)	192 (74.7%)	55 (67.9%)	
**On ARV's**				
Yes	274 (81.1%)	215 (83.7%)	59 (72.8%)	0.030^+^
No	64 (18.8%)	42 (16.3%)	22 (27.2%)	
**Missed clinic appointment in past 6 months**				
No	264 (78.1%)	200 (77.8%)	64 (79.0%)	0.821
Yes	74 (21.9%)	57 (22.2%)	17 (21.0%)	
**Perceived route of HIV acquisition**				
MTCT	183 (54.1%)	38 (56.4%)	38 (46.9%)	0.134
Other	155 (45.9%)	43 (43.6%)	43 (53.1%)	
**Peer Support group member**				
Yes	159 (47.0%)	137 (53.3%)	22 (27.2%)	<0.001^+^
No	179 (53.0%)	120 (46.7%)	59 (72.8%)	
**Alcohol use in last month**				
No	307 (90.8%)	238 (92.6%)	69 (85.2%)	0.044^+^
Yes	31 (9.2%)	19 (7.4%)	12 (14.8%)	

### Relationship status, marriage aspirations, disclosure and sexual behaviour of YLWH in central Uganda

Table 2 describes respondents’ relationship status, disclosure and sexual behaviour stratified by gender. Over half (64%) of all respondents interviewed had ever had a boy/girl friend (defined as companion of female (boyfriend) or male (girlfriend) with whom the individual is emotionally itimate with or without sexually relations). Among females, 70% had ever had a boyfriend compared to 47% among male, a difference that was statistically significant (chi square p value <0.001). However, at the time of the study 37% (126/338) were in a boy/girl relationship with no difference between genders. Of the 338 respondents interviewed, 83% (283/338) desired to get married in future. Among males, 95% desired to get married in future compared to 78% among females (fisher's exact test p value < 0.001). Among those who desired to get married (n = 283), majority, 79% (226) said the HIV status of their partners would be a key consideration when choosing a marriage partner. It is worth noting that among those whom the HIV status was a key consideration (n = 226), 50% (115) preferred to have a sero-negative spouse with no significant difference between males and females. Among those currently in a boy/girl relationship 69% (87/126) had not disclosed their HIV status to their partners. Only 37% (47/126) of those currently in a boy/girl relationship knew their partners HIV status. Forty percent (40%) of respondents who acquired HIV through other routes had disclosed their HIV status to their partners compared to 20% among perinatally infected respondents, a difference that was statistically significant (not shown). Overall 45% (150/338) had ever been sexually active. The mean age (S.D) of sexual initiation was 16.9 (±2.6). Ever being sexually active was significantly higher among females [55% (134/242)] compared to males [16% (16/96), chi square p value <0.001]. Despite the fact that a higher proportion of females had ever been sexually active, male initiated sex earlier at 15.4 years compared to females at 17.1 years, a difference that was statistically significant. Fifty five percent [55% (188/338)] of respondents had never been sexually active-primary abstinence and 24% [(269-188)/338] of those who had ever been sexually active had abstained for at least six month -secondary abstinence. Overall, 79% (269/338) of respondents were abstaining at the time of the study (defined as refraining from sexual intercourse for at least six months before the survey). A greater proportion of males (90%) compared to females (75%) were abstaining (chi square p value = 0.002). Among those who had ever been sexually active (n = 150), 53% used a condom at their last sexual encounter. Of the 20% (69/338) who were currently sexually active (reported sexual activity within 6 months before the survey), only 46% (32/69) reported consistently using condoms at every sexual encounter within the last 6 months. Among the 69 youth who were sexually active, 21 (30%) had multiple sexual partners.

**Table 2:** Relationship status, marriage aspirations, disclosure and sexual behaviour of YLWH by sex

Variable	Total (n = 338)	Females (n = 242)	Males (n = 96)	p value
**Boy/Girl Relationship status and Marriage aspirations**				
**Ever had a Boy/Girl Friend**				
Yes	217 (64.2%)	171 (70.7%)	46 (47.9%)	<0.001+
No	121 (35.8%)	71 (29.3%)	50 (52.1%)	
**Currently have Boy/Girl Friend**				
Yes	126 (37.3%)	97 (40.1%)	29 (30.2%)	0.09
No	212 (62.7%)	145 (59.9%)	67 (69.8%)	
**Desire marriage in future**				
Yes	283 (83.7%)	191 (78.9%)	92 (95.8%)	<0.001§+
No	55 (16.3%)	51 (21.1%)	4 (4.2%)	
**HIV status be considered in choice of partner (n = 283)**				
Yes	226 (79.9%)	154 (80.6%)	72 (78.3%)	0.642
No	57 (20.1%)	37 (19.4%)	20 (21.7%)	
**Preferred serostatus of marriage partner (n = 226)**				
Positive	112 (49.6%)	79 (51.2%)	33 (45.8%)	0.444
Negative	114 (50.4%)	75 (48.8%)	39 (54.2%)	
**Disclosure (n = 126-those currently in boy/girl relationships)**				
**Disclosed to partner**†				
Yes	39 (30.9%)	32 (33.0%)	7 (24.1%)	0.366
No	87 (69.1%)	65 (67.0%)	22 (75.9%)	
**Know partner's HIV status**†				
Yes	47 (37.3%)	35 (36.1%)	12 (41.4%)	0.605
No	79 (62.7%)	62 (63.9%)	17 (58.6%)	
**Sexual Behaviour**				
**Ever had sexual intercourse (n = 338)**				
No (Primary abstinence)	188 (55.6%)	108 (44.6%)	80 (83.3%)	<0.001+
Yes	150 (44.4%)	134 (55.4%)	16 (16.7%)	
**Mean (S.D) age at first sex**† **(n = 150)**	16.9(±2.62)	17.1 (±2.41)	15.4 (±3.85)	0.019+
**Condom use at last sexual encounter**† **(n = 150)**				
Used condom	80 (53.3%)	68 (50.8%)	12 (75.0%)	0.110§
Did not use condom	70 (46.7%)	66 (49.2%)	4 (25.0%)	
**Abstaining in past 6 months (n = 338)**				
Yes	269 (79.6%)	182 (75.2%)	87 (90.6%)	0.002+
No	69 (20.4%)	60 (24.8%)	9 (9.4%)	
**Consistent Condom use in past six months (n = 69)**				
Yes	32 (46.4%)	27 (45.0%)	5 (55.6%)	0.723§
No	37 (53.6%)	33 (55.0%)	4 (44.4%)	
**Multiple sexual partners** † **(n = 69)**				
No	48 (69.6%)	43 (71.7%)	5 (55.6%)	0.439§
Yes	21 (30.4%)	17 (28.7%)	4 (44.4%)	

+ statistically significant < 0.05

§ Fisher's test

† Frequencies of variable category do not add up to 338 because of skip patterns in questions

### Prevalence and factors associated with abstinence

Table 3 describes the unadjusted and adjusted prevalence proportion ratios (PPRs) of factors associated with abstinence. At unadjusted analysis, factors that were significantly associated with abstinence included HIV care site, sex, age, staying with parent, having a biological child, perceived route of HIV acquisition and taking alcohol. After adjustment, factors that remained statistically significantly associated with abstinence included younger age, having a biological child and taking alcohol at least once a month. The adjusted PPR was higher among younger respondents aged 15 to 19 years compared to those between 20 and 24 years (adj. PPR = 1.26 (1.08-1.46). The prevalence of abstinence was lower among respondents who had a biological child compared to their counterparts who did not have a biological child (adj. PPR = 0.77 (0.62-0.95) and among respondents who consumed alcohol at least once in the past month compared to those who did not consume alcohol throughout the month (adj. PPR = 0.31 (0.16-0.61). At unadjusted analysis, acquiring HIV through mother to child transmission was associated with higher PPR (unadj. PRR = 1.48, 95% CI (1.30-1.68) compared to borderline PPR after adjustment of 0.96, 95% CI (0.93-0.99).

**Table 3:** Prevalence of Abstinence, unadjusted and adjusted prevalence proportion ratios (PPRs) of factors associated with abstinence among youth aged 15 and 25 years in central Uganda

Characteristic	Abstinence% (n/N)	Unadjusted PPR (95% CI)	Adjusted PPR (95% CI)	p Value
**HIV care Site**				
Mildmay	82.1 (211/257)	1.15 (0.99, 1.33)	0.97 (0.94, 1.01)	0.098
Nsambya	71.6 (58/81)	1	1	
**Sex**				
Female	75.2 (182/242)	0.83 (0.75, 0.91)	1.01 (0.97, 1.05)	0.583
Male	90.6 (87/96)	1	1	
**Age group**				
15-19	92.7 (189/204)	1.55 (1.34, 1.79)	1.26 (1.08, 1.46)	0.003^+^
20-24	59.7 (80/134)	1	1	
**Marital Status**				
Single	81.8 (238/291)	1.24 (1.00, 1.53)		
Separated	66.0 (31/47)	1		
**Staying with Parent/Guardian**				
Yes	83.8 (237/283)	1.44 (1.14, 1.81)	1.05 (0.84-1.30)	0.652
No	58.2 (32/55)	1	1	
**Education Level**				
No formal education & Primary	73.2 (82/112)	0.88 (0.78-1.00)		
Secondary & Tertiary	82.7 (187/226)	1		
**Employment status**				
Employed	82.1 (220/268)	1.17 (1.00, 1.38)		
Unemployed	70.0 (49/70)	1		
**Have biological Children**				
Yes	53.9 (49/91)	0.60 (0.49, 0.73)	0.77 (0.62, 0.95)	0.015^+^
No	89.1 (220/247)	1	1	
**Route of HIV acquisition**				
MTCT	93.4 (171/183)	1.48 (1.30, 1.68)	0.96 (0.93, 0.99)	0.038^+^
Other	63.2 (98/155)	1	1	
**On ARVs**				
Yes	82.9 (227/274)	1.26 (1.05-1.52)		
No	65.6 (42/64)	1		
**Missed clinic appointment**				
Yes	71.6 (53/74)	0.88 (0.75, 1.02)		
No	81.8 (216/264)	1		
**Peer support group member**				
Yes	84.9 (135/159)	1.13 (1.02, 1.26)		
No	74.9 (134/179)	1		
**Take Alcohol**				
Yes	22.6 (7/31)	0.26 (0.14, 0.51)	0.31(0.16, 0.61)	0.001^+^
No	85.3 (262/307)	1	1	

## Discussion

This study demonstrates that a high proportion of YLWH had ever been sexually active although a considerable proportion where abstaining at the time of the study. The proportion abstaining in this study was considerable higher than was reported among adult's populations [14–16]. A high proportion of respondents in a boy/girl relationship had not disclosed their serostatus to their partners and only 37% of respondents who were currently in a relationship knew their partners’ HIV serostatus. Despite poor disclosure and knowledge of one's partner HIV status, condom use among sexually active respondents was low. Low condom use is a particular concern given that one quarter of sexually active YLWH had more than one sexual partner. HIV transmission in this population is a serious concern that calls for urgent attention. Younger youth between 15 and 19 years were more likely to abstain; similarly, a study at the paediatric infectious disease clinic in Uganda revealed that younger adolescents had a stronger belief in sexual abstinence [9]. Some governments and members of the international community have strongly promoted abstinence as an HIV prevention strategy for younger youth [17]; this approach has sometimes been criticised as being ineffective considering that many youth initiate sex early [18]. Similarly the findings of this study suggest that despite the high prevalence of abstinence among younger youth, mean age of sexual debut is at only 17 years. These findings suggest that advocating for abstinence-only programs for young people will not effectively control the epidemic. Abstinence should therefore be promoted alongside other HIV prevention strategies that address prevention needs of sexually active YLWH. The prevalence of abstinence was higher among respondents who did not consume alcohol. Another study among unmarried youth in the general Rwanda population reported that alcohol use negatively influenced primary abstinence [19]. The 2006 Uganda Demographic and Health Survey (UDHS) shows that engaging in sex under the influence of alcohol impairs judgement thus increasing risky sexual behaviour [20]. Alcohol intake is also associated with poor adherence to antiretroviral medication and reduced immune response to the HIV virus [21]. Furthermore, a study conducted among sero-positive youth in Tanzania showed that alcohol use hindered condom use [22], similar finding were reported from an adult sero-positive population in Uganda [16]. In addition to decreasing the practice of abstinence available literature elaborates other negative effects of alcohol consumption on HIV prevention and treatment outcomes.

Alcohol intake should therefore be discouraged among YLWH for both HIV prevention and to improve treatment outcomes. Enforcing the law against alcohol consumption for youth below 18 years may avert the use of alcohol generally among younger youth. Health education should highlight the negative effects of alcohol intake and youth addicted to alcohol should be provided with psychosocial support to stop the addiction. Considering the far-reaching effects of alcohol consumption, behavioural change messages educating HIV sero-positive communities about the negative effects and consequences of alcohol consumption should be developed. More than three quarters of the study population desired to get married and have children in the future. This raises questions on their choice of sexual/marriage partners and its implication for HIV transmission. The vast majority reported that the HIV status of their marriage partner would be a key consideration. Half of these respondents preferred sero-negative marriage partners. In another study among YLWH between 15 and 19 years, one third of respondents preferred to have negative partners [10]. It is important to note that people living with HIV have a right to choose a sexual partner and enjoy a fulfilling sexual life and the right to intimacy, to have children, and to love. However, along with these rights, sero-positive people have the responsibility not to put anyone at risk of contracting HIV [23]. Health education and counselling should highlight these rights while emphasizing their responsibility to stop the further transmission of infection through partner sero-sorting. Behavioural change communication should highlight safe sex measures for prevention of HIV transmission and re-infection depending on the sero-status of one's partner. The preference for sero-negative partner could explain why 36% of YLWH who knew their partner's status were in discordant relationships. The proportion in discordant relationships is probably higher since a considerable proportion of YLWH did not know the serostatus of their sexual partners. This study emphasizes the increasing need of HIV prevention measures among individuals in discordant relationships. Discordant couples should continually be counselled on safe sex practices and the importance of couple HIV counselling and testing should be emphasized to individuals who do not know their partners HIV status.

Despite the crucial role disclosure plays in HIV prevention, over two thirds of respondents with steady partners had not disclosed their serostatus to their partners and only 37% of respondents who were currently in a relationship knew their partners’ HIV serostatus. Disclosure in this study was lower than the 86% reported in a nationally representative Ugandan sample of people aged between 15 and 59 years [24] and the 69% among HIV positive adult respondents in Eastern Uganda [25]. Disclosure is a key HIV prevention component that influences sexual behaviour among HIV infected individual and their partners [26]. Inadequate disclosure in this study is a critical issue that needs to be addressed because disclosure is the basis for sexual behaviour decision making that directly affects HIV prevention. Perinatally infected youth seem to be disproportionally greater disclosure challenge. Behavioural change communication messages for YLWH, peer education and psychosocial counselling should emphasize the critical importance of disclosure for sero-positive individuals. Empowering perinatally infected youth with disclosure skills also needs to be given greater priority. Consistent condom use among sexually active youth was very low at 38%. It could be urged that the low prevalence of consistent condom use is due to the fact that majority of respondents were females since women are more likely to report unprotected sex compared to men [27]. There were no gender differences in frequency of condom use. Similarly, a study among YLWH reported consistent condom use at only 34% [28]. The proportion of YLWH consistently using condoms was lower than the 65% consistent condom use reported in a study among sero-positive adults at the Joint Clinical Research Centre in Kampala [29]. Although not directly comparable, poor condom use has been reported from nationally representative samples of HIV infected people in Uganda, Kenya and Malawi [24, 30]. Qualitative study findings indicate that adults living with HIV in central Uganda find it challenging to consistently use condoms [31]. Qualitative studies exploring the challenges to condom use is this population should be conducted in order to develop tailor-made interventions. Twenty three percent of sexually active individuals reported having more than one sexual partner in the previous six months. Consistent condom use with irregular sexual partners was reported at only 32%. Among public hospitals in Ethiopia, 10% of participants had multiple sexual partners and having multiple partners was associated with unprotected sex [32]. The high multi-partnership rate in our study coupled with the low condom use and inadequate disclosure poses a serious threat to HIV prevention and has the potential to fuel transmission of the epidemic. The findings of this study should be interpreted in light of some limitations. The findings of this study are based on self-reported sexual behaviour which has been biased in some settings [33]. To minimise this bias several validating questions were included in the questionnaire. Questions on each outcome were asked in a sequential manner, starting with the most recent to ensure that correct information was collected and to minimise recall bias.

## Conclusion

A big proportion of single YLWH were currently abstaining, despite this nearly half had ever been sexually active. Condom use at last sexual encounter was reported by only half of the respondents and disclosure among respondents in relationships was inadaquate. These findings highlight that many single YLWH engage in bahaviours that place their sexual partners at risk of HIV infection. Alcohol consumption hindered abstinence and younger youth were more likely to abstain. Important to note is that a significant proportion of unmarried YLWH aspired to have negative marriage partners in future. This raises concerns for HIV prevention among discordant couples considering the current challenges influencing consistent condom use among such couples [34]. These findings add to the existing evidence for the need for positive prevention programming in Uganda. Exploratory research investigating strategies to improve HIV prevention among seropositive individuals should be conducted. Additionally considering that many young people infected with HIV now have the fortunate opportunity to live a normal and healthy life they should be empowered to make reproductive health choices that avert HIV transmission risk inorder to control the epidemic.

### What is known about this topic

- A significant proportion of YLWH initiate sex during adolescence despite the advocacy for abstinence as an HIV prevention strategy for young people. Despite this, there is inadequate disclosure and condom use;
- Majority of adolescents living with HIV desire to have children and family. This raises concerns about their partner preferences and it's implication on HIV transmission.

### What this study adds

- Nearly half of the respondents had ever been sexually active. Only one third of YLWH who had a boy or girlfriend knew their partners’ serostatus. Additionally, one third of those currently sexually active had more than one sexual partner. Consistent condom use with irregular sexual partners was only 32%. The high multi-partnership rate coupled with the low condom use and inadequate disclosure among YLWH in this study raises serious concern of the potential high risk of HIV transmission in this population;
- We established that half of YLWH preferred sero-negative marriage partners. The preference for sero-negative partners possibly explains why one third of YLWH who knew their partner's status were in discordant relationships. This emphasizes the increasing need for partner sero-sorting counselling and HIV prevention measures for discordant couples.
